# The utility of ultrasound and computed tomography in the assessment of carotid artery plaque vulnerability–A mini review

**DOI:** 10.3389/fcvm.2022.1023562

**Published:** 2022-11-16

**Authors:** Aniruddha Singh, Usama Nasir, Jared Segal, Tayyab Ali Waheed, Muhammad Ameen, Hamza Hafeez

**Affiliations:** ^1^College of Medicine, University of Kentucky, Lexington, KY, United States; ^2^Tower Health, West Reading, PA, United States

**Keywords:** plaque vulnerability, carotid ultrasound, CEUS, unstable plaque, CT

## Abstract

As the burden of cardiovascular and cerebrovascular events continues to increase, emerging evidence supports the concept of plaque vulnerability as a strong marker of plaque rupture, and embolization. Qualitative assessment of the plaque can identify the degree of plaque instability. Ultrasound and computed tomography (CT) have emerged as safe and accurate techniques for the assessment of plaque vulnerability. Plaque features including but not limited to surface ulceration, large lipid core, thin fibrous cap (FC), intraplaque neovascularization and hemorrhage can be assessed and are linked to plaque instability.

## Introduction

The most frequent cause of coronary and carotid artery disease is atherosclerosis. Plaque with high-risk features are characterized as “vulnerable” and are associated with a greater probability of neurologic and cardiovascular events (1). There is evidence that not only luminal narrowing but plaque morphology plays a vital role in characterizing such vulnerable plaques (2). Carotid plaques may rupture and lead to transient ischemic attacks or ischemic strokes (3). There are currently no therapies for vulnerable plaque beyond treatment with statins, as stenting and endarterectomy are recommended for the treatment of symptomatic patients with high stenosis. However, studies have shown that carotid plaques with high risk features albeit <50% stenosis may be linked to cryptogenic ischemic strokes (4). The dynamic nature of atherosclerotic plaque and its potential consequences has led researchers to focus on non-invasive methods for their early detection and identification. Duplex ultrasound is simple, inexpensive, and can be used to assess the morphology and degree of carotid stenosis. Determination of carotid plaque morphology including but not limited to ulceration, plaque area, intraplaque hemorrhage (IPH), and plaque echogenicity may be useful in identifying patients with asymptomatic carotid disease who are at higher risk of adverse events (2). CT including both dual source CT (DSCT) and multidetector CT angiography (MDCTA) has emerged as a reliable tool in the assessment of vulnerable plaque as well. This review summarizes the utility of ultrasound and CT in the evaluation of the vulnerable carotid plaque. Early detection *via* these modalities can prevent neurologic and cardiovascular events.

### Ultrasound and the carotid vulnerable plaque

Carotid ultrasound has been utilized to predict the risk of cerebrovascular disease (CVD). Traditionally, measuring the carotid intima media thickness (CIMT) has been verified as an important estimator of CVD (5). However, potential pitfalls in the assessment of CIMT has made it fall out of favor. In contrast, recent studies have demonstrated the assessment of carotid plaque itself as a more accurate means of assessing the risk of CVD (6–8). Inflammatory changes in an unstable plaque have shown to contribute more to CVD events than direct extension of atherosclerosis (9). Ultrasound is a safe and non-invasive method to assess plaque vulnerability. Carotid plaque evaluation *via* ultrasound should include a detailed assessment of the number of plaques, plaque thickness/height, plaque area, surface features, neovascularization, and when possible, a 3D assessment of the entire vessel involved (7).

Various ultrasonographic methods can be used to assess atherosclerotic plaques including real time ultrasound, doppler ultrasound, non-doppler flow evaluation methods, optimal ultrasound, Contrast enhanced ultrasound (CEUS), and shear wave elastography (10).

### Plaque surface irregularities

Plaque ulceration has long been shown to correlate with neurological symptoms and the occurrence of stroke (11). The detection of plaque ulceration is superior via CEUS as opposed to B-mode or color flow doppler sonography due superior sensitivity (88% compared to 29%) (12). In individual studies, B-mode and color flow doppler have shown sensitivities and specificities of 35.7–85.7% and 75%–81.3% respectively. The latter lacks sensitivity in high-grade stenosis (13).

Echo-intensity serves as a marker of surface morphology. Uniform echo intensity corresponds to a smooth and regular surface whereas a non-uniform pattern and mixed echo-intensities indicate surface heterogeneity (14). Criterion used to classify ulceration vary, largely the projection of a column of microbubbles within an atherosclerotic plaque of 1 × 1 mm or more has shown a high sensitivity (15, 12)

**FIGURE 1:**
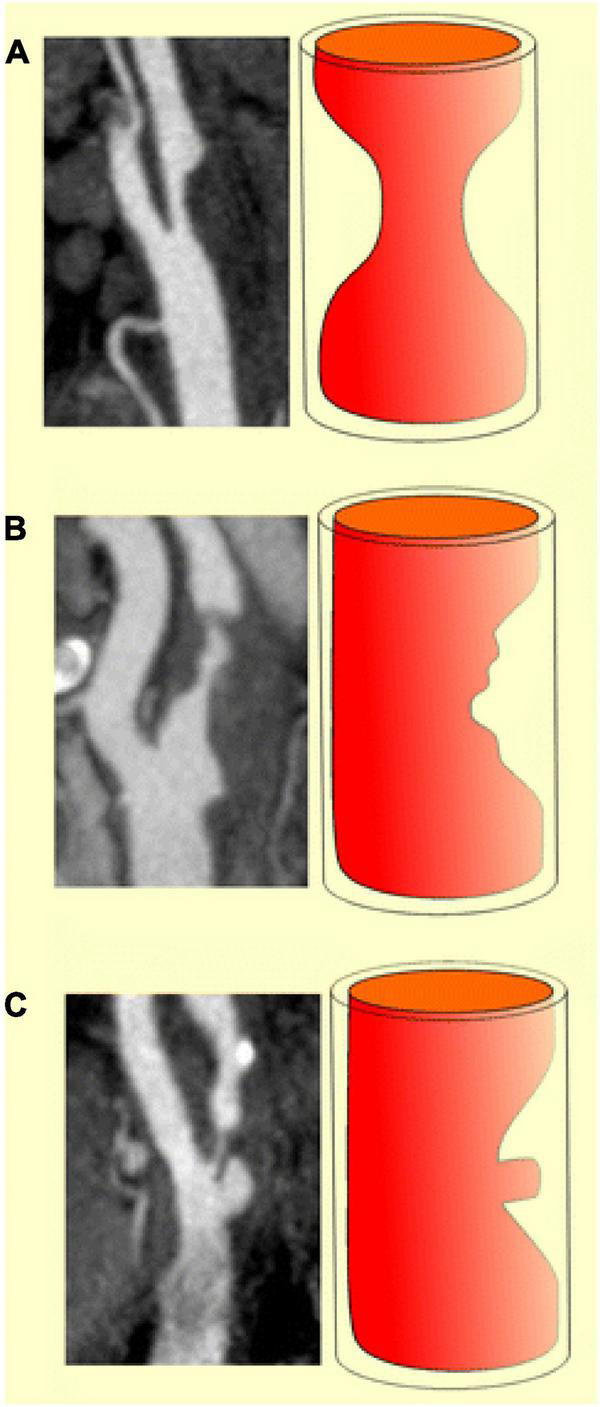
Diagrammatic representations and MDCTA images illustrating the classification of carotid plaques based on their surface morphology as smooth **(a)**, irregular **(b)** and ulcerated **(c)** (Content copied in its original form from reference (19) under Creative Commons Attribution 4.0 International License).

Plaque surface has broadly been classified over the spectrum of smooth, irregular, and ulcerated (Figure 1) and correlates with the risk of embolic strokes (16, 17). Smooth plaques lack surface irregularities, irregular plaques have surface irregularities ranging from 0.3 to 0.9 mm without ulceration (18), while ulcerated plaques have at least one focal cavity measuring from 1 to 2 mm in depth which leads to exposure of the underlying necrotic core (17).

### Plaque echogenicity

Echogenicity of the plaque is directly related to the amount of calcification and fibrous tissue and inversely related to the lipid content of the plaque. IPH is directly related to lipid content and inversely related to the amount of fibrous tissue in the plaque. Therefore, the association between IPH and a high lipid content may support the theory of the lipid-rich plaque being more prone to rupture (20–24).

Echogenicity of a plaque is graded across type 1 to 5 based on the Gray-Weale-Nicolaides (GWN) classification (25, 26):

Type 1: Homogeneously translucent plaque which is difficult to distinguish from fluid inside the vessel. Plaque primarily composed of lipids and necrotic material.
Type 2: Echo lucent plaque with the presence of single calcifications not exceeding 25% of plaque volume or 20−25% of plaque size.
Type 3: Predominantly echogenic plaque−calcifications constitute up to 50% of plaque structure.
Type 4: Uniformly echogenic with greater than 50% uniform calcification.
Type 5: Heavily calcified plaque.

### Thickness of the fibrous cap, size of the lipid necrotic core

Thinning and rupture of the fibrous cap (FC) is sentinel in plaque instability. Rupture is common in plaques with FC thickness less than 0.065 mm (27–30). Neovascularization found in the FC especially in the medial and lateral corners can become leakage sites of blood vessels through the accumulation of inflammatory cells. This adds to the vulnerability of the plaque (30). Thin or ruptured FC has also been linked to plaque ulceration which is a known marker of plaque instability. In addition to thickness, echogenicity of the cap is also important. A thin FC defines the plaque as thin cap atheromatic plaque (TCAP) which makes the plaque vulnerable to rupture (26).

Lipid core accounting for 40% of the plaque volume makes it prone to rupture (13). Echo lucent plaques are lipid rich while echogenic plaques are fibrin rich with calcification. Plaque echogenicity can be graded from one to four as described previously. Echo lucent plaques have a higher association with CVAs (31, 32).

### Carotid neovascularization

Atherosclerosis within the plaque leads to local hypoxia promoting neovascularization and vessel wall injury. Vessel wall injury in turn leads to inward growth of the vasa vasorum leading to further neovascularization. This immature neovascularization leads to increased vessel wall density. As the vascularity grows, the size of the core grows which stretches the FC thin. This is referred to as thin fibrous cap atheroma/thin cap atheroma plaque (TFCA/TCAP). The microvessels lack wall integrity, bleed and lead to IPH which compromises stability of the plaque (10, 33–35). IPH has been correlated with increased incidence of CVD (36).

**FIGURE 2:**
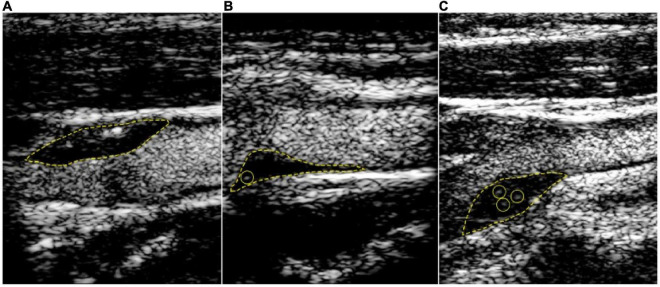
Contrast-enhanced carotid ultrasound for the detection of plaque neovascularization. Content copied in its original form from Mantella et al. with permission (42). Carotid intraplaque neovascularization scoring method. Representative contrast-enhanced ultrasound images of carotid plaques. **(A)** demonstrates a plaque score of 0, no visible microbubbles within the plaque; **(B)** demonstrates a plaque score of 1, minimal microbubbles confined to peri-adventitial area; **(C)** demonstrates a plaque score of 2, microbubbles present throughout the plaque core. The yellow dotted line outlines the plaque lesion. Yellow circles depict intraplaque contrast microbubbles.

Studies have shown consistency of CEUS in the detection of neovascularization (37, 38). CEUS uses ultrasonographic contrast agent (UCA) consisting of micro bubbles which reflect ultrasound waves as harmonic frequencies back toward the transducer. The contrast agents are composed of small microbubbles that remain intra-arterial and can pick up microvasculature in the adventitia and the core of the plaque. The microbubbles give off signals back to the transducer which are reflective of the microvasculature. This intraplaque enhancement can represent IPH, immature leaky vasa vasorum and neovessels in vulnerable plaques. Signal intensity may correlate with the density of the microvasculature and be directly related to the vulnerability of the plaque (39, 40). Intraplaque enhancement has been graded for qualitative assessment. Grade 1 (mild) no intra plaque enhancement, Grade 2 (moderate) enhancement of the plaque shoulder and adventitia, or Grade 3 (severe) intraplaque enhancement (40, 41). Grade 4 has also been used which involves more extensive infiltration into the plaque body (Figure 2).

### Computed tomography imaging of carotid artery vulnerable plaque

Traditionally, MRI has been considered the imaging of choice in evaluating high risk plaques. However, recent advances in CT have made it a reliable resource for detecting plaque vulnerability. MDCTA and DSCT are the two most widely available techniques utilized for plaque assessment. Compared to conventional CT which utilizes individual slices, MDCTA acquires volume data, has much higher data acquisition speed and therefore allows for a much higher spatial resolution. This allows for better visualization of the tissue components of a vulnerable plaque compared to MRI (43, 44).

Although MDCTA’s wide availability makes it a convenient resource, it does have noticeable difficulty in differentiating between plaque calcium and luminal contrast (38). These restrictions are overcome with the DSCT technology that visualizes distinct radiodensities in a carotid plaque. Clear distinction between luminal contrast and the plaque body is achieved with DSCT. Unlike MDCT, DSCT is not as widely available but provides greater temporal resolution compared to MRI (13, 45).

Multidetector computed tomography and dual source computed tomography also help assess details of a soft plaque. Soft plaque is a combination of IPH, lipid-rich necrotic core (LRNC) and fibrous elements. On CT, soft plaque is generally defined as a low attenuation plaque with roughly <60 Hounsfield units (HU), whereas fibrous tissue is considered between 60−130 HU and >130 HU is considered calcified plaque. Soft plaque is associated with a threefold increase in cerebrovascular events (46), hence it is increasingly important for imaging techniques to efficiently discover soft plaque of the carotid arteries.

### Computed tomography evaluation of intraplaque-hemorrhage

Intra-plaque hemorrhage is a critical event preceding an acute ischemic events. Neo-vasculature that has invaded into the plaque can rupture and cause IPH. Factors contributing to micro hemorrhaging include diabetes, metabolic derangements, etc. (47).

Recent research has suggested that CT can identify these high risk features in a plaque, despite MRI having been considered the foremost imaging modality for IPH in the past. One study by Saba et al. in 2018 suggested that Hounsfield units <25 on CT consistently identified the presence of IPH. Utilizing this information, a retrospective study published in 2019 by Saba et al. evaluated components and subcomponents of plaque volume and IPH in 200 carotid arteries that underwent CTA. Their research suggested that Hounsfield units <25, which represented IPH, showed a statistically significant association with the presence of cerebrovascular events in patients (48).

### Computed tomography evaluation of the lipid rich necrotic core

Differentiating between LRNC and IPH on CTA can be challenging as both have low attenuation (<60 HU). However, IPH is considered to have lower Hounsfield unit values on average than LRNC, 18 HU compared to 63 HU, respectively. In general, low attenuation still represents the presence of high risk soft plaque and differentiating between IPH and LRNC on CTA may not have a clinical importance (45).

### Computed tomography evaluation of the fibrous cap

Assessment of FC integrity plays a critical role in differentiating between low and high risk plaques. Low risk plaques have an intact FC, thinned FC is related with mildly increased risk of rupture, while a fissured FC overlying a large LRNC carries a very high risk of plaque rupture. Once rupture occurs, the thrombogenic subendothelial plaque and its matrix are exposed to intraluminal blood flow and can lead to thromboembolism.

Saba et al. in 2013 showed a correlation between fissured FC and contrast enhancement on MDCTA. Forty-seven symptomatic patients underwent MDCTA scans and contrast enhancement of the plaques were analyzed. Patients then underwent carotid endarterectomy followed by histologic analysis of the plaque to evaluate for fissured FC’s. Of the forty-seven patients, twelve were found to have fissured FC’s, and 92% (11/12 patients) of fissured FC’s had contrast enhancement on CTA. Of the non-fissured FC’s, 69% (24/35 patients) also had contrast enhancement, however, to a much lesser degree (22.6 HU as opposed to 12.9 HU) (49). This suggests that MDCTA can evaluate for vulnerable plaque using contrast enhancement on MDCTA.

### Surface morphology

Ulcerated plaque surfaces, defined as a cavity of >1 mm, are most concerning on VT imaging. MDCTA has a higher sensitivity and specificity than digital subtraction angiography and ultrasound at detecting these ulcerations (44).

## Discussion

**FIGURE 3:**
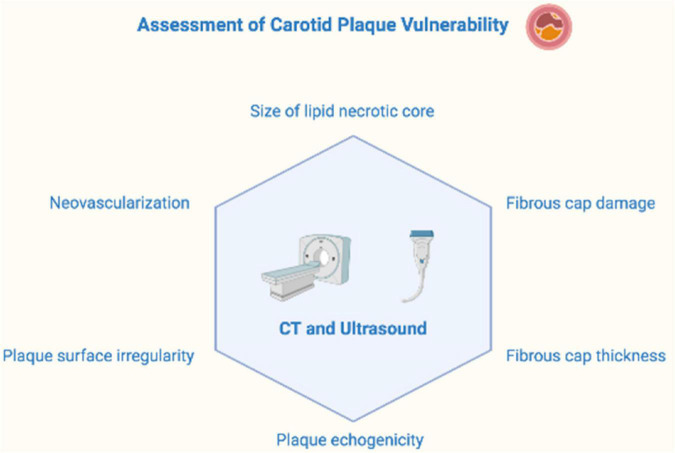
Features assessed in the qualitative analysis of plaque vulnerability.

The emerging concept of plaque vulnerability has been well documented in the recent years. Assessment of plaques for their vulnerability as opposed to traditional vascular stenosis can better quantify the risk of embolic events. The current review summarizes the sentinel features of the vulnerable plaque, and outlines the role of carotid ultrasound and CT in the identification of such vulnerable features (6, 7). Figure 3 shows a graphical summary.

The connection between vulnerable plaque and vulnerable patient was described in the SHAPE taskforce report. Iliofemoral and carotid atherosclerosis are CHD risk equivalents. These predict atherosclerosis in other vascular beds and should be treated aggressively (50) In asymptomatic patients with carotid atherosclerosis, the utility of revascularization remains to be proven. However, individual risk factor assessment including features of plaque vulnerability may identify a high risk patient for a near term event. Therapeutic strategies focusing these patients can decrease the burden of palliative care for CVD.

Ultrasound serves as the first line modality for many vascular studies. In comparison to CT and MRI, CEUS can be used for the assessment of thrombus vascularity via real-time and continuous scanning (51). Compared to standard carotid intima-media thickness (cIMT), the presence of plaque on carotid ultrasound is a superior predictor of future vascular events.

Ultrasound has a lower overall cost, and is relatively safe compared to CT modalities due to the lack of iodinated contrast media. One of the major limitation of ultrasound and CEUS is subjective interpretation by the investigator which is constant along the spectrum of ultrasound based studies. Secondly, due to the specialized nature of CEUS and its contrast agents, wider availability is yet to be attained. In addition, CEUS requires superior software modalities to better process impulses received by the transducer. Contrast medium consists of microbubbles filled with high molecular weight gas which can rarely cause headaches, injection site bruising, pain, and paresthesia’s. (40)

Limitations of CT imaging include beam hardening artifacts which can be common in MDCTA secondary to calcification in the arteries and plaques. In comparison to ultrasound, contrast agents used in CT carry the risk of hepatotoxicity, renal toxicity, and allergic reactions. In comparison, contrast agents used for CEUS contain microbubbles which are mainly metabolized by respiration and are safer (44, 51). In addition to being more expensive, CT also carries the risk of ionizing radiation.

## Conclusion

Plaque vulnerability is a superior marker in predicting future risk of cerebrovascular events. CEUS and CT assessment are emerging as easy non-invasive tools for quantitative and qualitative assessment of plaque vulnerability. These modalities can identify surface and intraplaque irregularities which are markers for plaque instability. The use of these modalities should be increased in routine carotid plaque assessment.

## Author contributions

UN took responsibility for all aspects of the reliability and freedom from bias of the data presented and the discussed interpretation. UN, MA, JS, HH, and TW contributed equally toward manuscript preparation, literature search, getting references, and revising the manuscript. All authors contributed to the article and approved the submitted version.
